# Evaluating the Cellular Roles of the Lysine Acetyltransferase Tip60 in Cancer: A Multi-Action Molecular Target for Precision Oncology

**DOI:** 10.3390/cancers16152677

**Published:** 2024-07-27

**Authors:** Nazanin Zohourian, Erin Coll, Muiread Dever, Anna Sheahan, Petra Burns-Lane, James A. L. Brown

**Affiliations:** 1 Department of Biological Science, University of Limerick, V94 T9PX Limerick, Ireland; zohourian.nazanin@ul.ie (N.Z.);; 2 Limerick Digital Cancer Research Centre (LDCRC), Health Research Institute (HRI), University of Limerick, V94 T9PX Limerick, Ireland

**Keywords:** breast, cancer, Tip60, Kat5, KAT, HAT, TNBC, inhibitor, epigenetic

## Abstract

**Simple Summary:**

Individualized medicine means understanding how each tumor is different from normal cells and how each tumor is different from other tumors, including profiling mutations, non-mutational epigenetic changes, and differences in gene expression. This allows the discovery of key processes each tumor absolutely depends on for survival and growth, which are intrinsic weaknesses. This profiling means selecting treatments to specifically target each tumor’s survival-dependent pathways, killing them. Tip60 is a master controller of processes that maintain genome stability and signaling regulating gene expression. While disrupted in many cancers, Tip60 is essential for cell survival, and inhibiting Tip60 kills tumors. While we understand some key aspects of the molecular roles Tip60 plays, much more remains to be discovered. A more complete understanding of the diverse roles and functions of Tip60 in cancer, and how targeting Tip60 kills cancer cells, will lead to better treatments for patients and increased survival.

**Abstract:**

Precision (individualized) medicine relies on the molecular profiling of tumors’ dysregulated characteristics (genomic, epigenetic, transcriptomic) to identify the reliance on key pathways (including genome stability and epigenetic gene regulation) for viability or growth, and then utilises targeted therapeutics to disrupt these survival-dependent pathways. Non-mutational epigenetic changes alter cells’ transcriptional profile and are a key feature found in many tumors. In contrast to genetic mutations, epigenetic changes are reversable, and restoring a normal epigenetic profile can inhibit tumor growth and progression. Lysine acetyltransferases (KATs or HATs) protect genome stability and integrity, and Tip60 is an essential acetyltransferase due to its roles as an epigenetic and transcriptional regulator, and as master regulator of the DNA double-strand break response. Tip60 is commonly downregulated and mislocalized in many cancers, and the roles that mislocalized Tip60 plays in cancer are not well understood. Here we categorize and discuss Tip60-regulated genes, evaluate Tip60-interacting proteins based on cellular localization, and explore the therapeutic potential of Tip60-targeting compounds as epigenetic inhibitors. Understanding the multiple roles Tip60 plays in tumorigenesis will improve our understanding of tumor progression and will inform therapeutic options, including informing potential combinatorial regimes with current chemotherapeutics, leading to improvements in patient outcomes.

## 1. Introduction

**Figure 1:**
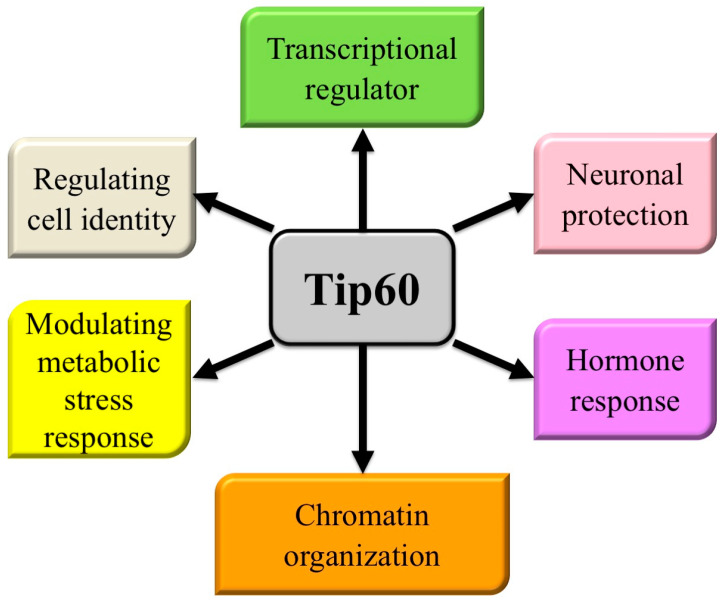
Pathways regulated by Tip60. Cellular processes in which Tip60 has a significant known role.

Modern molecular medicine (individualized or precision medicine) relies on profiling tumors (including using genomic, epigenetic, transcriptomic, or proteomic fingerprinting) to identify tumor cells’ reliance on key pathways for survival or growth, and then uses small molecule inhibitors (often inhibiting the activity of a single molecule) or biologics to disrupt these essential survival-dependent pathways [1,2,3]. The specificity of these treatments is enhanced by using biomarkers paired to the inhibitor to select sensitive patient cohorts (by excluding patients that are treatment insensitive), which leads to improved patient outcomes. The next generation of molecular medicines will target molecules that are key regulators of multiple processes, both improving their efficacy and enhancing their range of use to include more tumor types/profiles and other diseases [4,5,6]. One such multi-process molecule is Tip60 (Tat-interactive protein 60-kDa) (Figure 1), a member of the MYST family of acetyltransferases (one of five acetyltransferases families [7,8,9,10]).

## 2. Tip60

**Figure 2:**
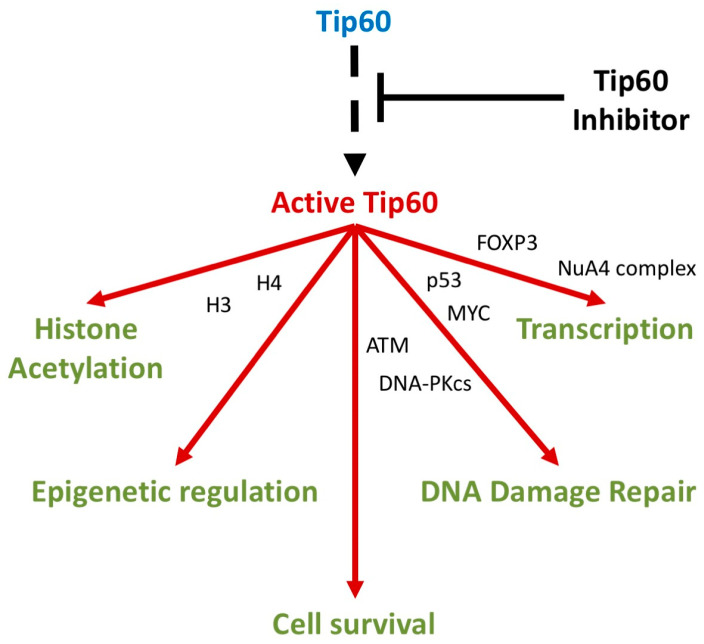
Key molecular processes regulated by Tip60 activity. Molecular signaling cascades (arrows indicate key pathways/cascades) where Tip60 has a significant known molecular role. Key Tip60-interacting proteins indicated (proteins between arrows indicate overlapping roles in adjacent processes).

Tip60 is a versatile lysine acetyltransferase, acetylating the ε-amino groups of lysine residues in various proteins [11]. Significant Tip60-dependent processes include regulating genome stability, transcriptional and epigenetic regulation, immunoregulation, and membrane receptor expression (altering receptor pathway activity) (Table 1, Figure 2) [12,13]. Tip60 activity governs diverse cellular activities required for cell survival through modifications to key substrates including histones 2A, 3, and 4 (facilitating chromatin remodeling) and non-histone proteins (including p53, MYC, and ATM) [14,15].

Table 2 lists many known Tip60-interacting proteins, including their described cellular functions/signaling pathways [16]. An issue worth highlighting is that much of the key work exploring the molecular roles of Tip60 has relied on using either gene knockouts (KO) or gene expression knockdown (KD) (often small interfering RNA) systems. These methods produce an asynchronous pool of Tip60 KO/KD cells (most often in cancer cell lines) which are in different stages of apoptosis, as loss of the essential Tip60 gene/protein induces cell death. This complicates the analysis of any roles of Tip60, as the Tip60-dependent signaling investigated is intrinsically entangled with the induced apoptotic signaling [17]. Additionally, many experiments have focused on very early time points in Tip60-dependent signaling cascades (often in DNA damage response pathways), which must be taken into account when reviewing our understanding of Tip60-mediated roles [8,18].

**Table 1:** Cellular roles of Tip60.

Function	Molecular Process
Regulating cell identity	Stem cell identity [18,19,20,21]
	Enhancing Treg cell induction [22,23]
Transcriptional regulator	Transcription [13,24]
Modulating metabolic stress response	Cell survival [13,25]
Hormone response	AR signaling response [26,27]
Genome stability/chromatin remodeling	DNA damage repair [14,24]
	Transcriptional regulation [28]
Neuronal protection	Neuronal cell function [16,20]
Cell cycle	Regulating Mad1/2 expression [13,29]

**Table 2:** Tip60 interactive proteins.

Protein Name	Signaling Pathway
FOXP3	Transcription
APBB1 (Fe65)	
C/EBP α	
Interleukin-9 receptor	
STAT3	
HDAC7	
KLF4	
ATXN1	
Epc1	
Epc2	
MBTD1	
Gas41/YEATS4	
MYC	
RELA/p65	
SOX9	
ATM	DNA damage response
P53	
SIRT1	
TRRAP	
FAM135B	
ATF2	
p400	
MOF	
BAF53a	
ANP32E	
RNF8	
UHRF1	
FLJ10914/MRGBP	
MORF4L1	
TRIM29	
ACTL6A	
TRCp120	
P300	Enhancing Treg cell induction
USP7	
FOXP3	
Androgen receptor	AR signaling response
HDAC1	
RuvBL1	Tip60 complex assembly
RuvBL2	
ING3	Apoptosis
APP	
YL-1	Chromatin remodeling
UHRF1	
P400	
HDAC9	
MORF4L2	
JAZF1	
DMAP	DNA replication
Mdm2	Regulation of Tip60
Cul3	
ATF3	
UHRF2	

Furthermore, despite the many roles of Tip60 in different cellular processes, numerous studies have had a single-role focus, which then fails to fully profile the multi-functional activity and/or networked effects of Tip60 loss on the system; i.e., a study focused on genome stability may not explore the effects of Tip60 loss on transcriptional regulation.

## 3. Tip60-Modulated Transcriptional Regulation

Due to the key significance of histone modifications in regulating chromosome structure and transcription, the role of Tip60 in the regulation of transcriptional processes has been explored [12,24]. The direct role of Tip60 in regulating the expression of several genes has been described (reviewed in Table 3). As the catalytic subunit of the NuA4 (nucleosome acetyltransferase of H4) complex, Tip60 plays a vital role in transcriptional activation and is a co-activator for numerous transcription factors and demonstrates binding (along with other Tip60 complex members) to promoters regulated by E2F or c-myc [27,30]. Tip60, concurrently with other NuA4 subunits like p400, is involved in facilitating p53-mediated transcription [31]. Furthermore, Tip60, as a transcriptional co-activator of p53, increases the activation of *p21* and *puma*, which have a role in growth arrest and apoptosis. In response to DNA damage, Tip60 acetylates p53K120 (within p53’s DNA-binding domain), regulating the selection of promoters and ultimately altering the cellular response from cell-cycle arrest to apoptosis [32]. The identification of p53K120 acetylation by Tip60 is important because it represents one post-translational modification of p53 linked to a residue which is frequently mutated in cancer (promoting tumorigenesis) [33]. While it is known that loss of Tip60 induces apoptotic cell death, which primarily appears to result from increased DNA damage induced genome instability and triggers apoptosis, it remains likely that the dysregulation of Tip60-dependent transcriptomic and/or epigenetic pathways will correspondingly contribute significantly to Tip60-dependent cell survival [18,34,35].

## 4. Tip60-Modulated Epigenetic Regulation

Tip60 has been identified as an epigenetic regulator through its role as a co-activator or corepressor of transcription factors and through its role in chromatin remodeling by histone acetylation [28,35,42]. As previously highlighted (Section 2), many approaches investigating Tip60 were conducted on an induced background of apoptotic signaling; as such, the epigenetic effects of Tip60 loss (alone and without apoptotic signaling) are poorly understood. Furthermore, it is likely that these Tip60-dependent epigenetic roles of Tip60 are tissue-specific (as is seen with the known cell-specific immunoregulatory and stem cell identity roles). Interestingly, Tip60 activity has been shown to be important for cognition-linked processes in brain tissue, where Tip60-mediated transcriptional regulation mediates cognitive function (in *Drosophila*) [43]. Together, the roles of Tip60 in chromatin remodeling and transcription support the need to use precise and tunable Tip60 inhibitors to better understand the epigenetic roles of Tip60 in each tissue type (without the confounding effects of apoptosis induction due to the genetic/transcript loss of Tip60) and as potential epigenetic therapeutics for the treatment of cancer and other diseases, including neurological and immune disorders.

## 5. Tip60-Modulated Immunoregulation

A key element regulating the immune system response to tumors is mediated through T-regulatory (Treg) cells, with Treg cells characterized by their expression of the transcription factor Foxp3 expression, which is Tip60-dependent [44,45,46]. It has been demonstrated that the activity of Tip60 significantly influences this immunoregulation through the Foxp3-driven modulation of regulatory T cells (Tregs). The Tip60–Foxp3 interaction enhances both the stability and transcriptional activity of Foxp3, where Tip60 acetylates Foxp3, preventing its polyubiquitination and degradation, ensuring increased protein levels [47], which promotes the suppressive Foxp3 Treg functions, helping drive tumor immunity [46,48,49,50,51]. This indicates that targeting Tip60 would have a significant effect on tumors’ immunological profile, mediated through Treg cells, with a strong potential for beneficial therapeutic effects in oncology and autoimmune disease treatments [52,53]. While Tip60 plays a key role in mediating the immune response through transcriptional activities, other key roles include more direct functions in protecting genome stability.

## 6. Tip60-Regulated Genome Stability

Cells protect genomic integrity through many mechanisms, and the DNA damage response (DDR) pathway is essential for repairing double-strand DNA breaks (DSB). The DSB response is regulated by the apical kinase ATM, and ATM activation requires acetylation by the lysine acetyltransferase Tip60, positioning Tip60 as a master regulator of the DSB response [14,54,55]. Tip60 contributes to the DDR through two key molecular pathways: DSB chromatin remodeling (involving Nu4A) and through Tip60-dependent-activation of ATM. Tip60 is recruited to damaged sites, acetylating lysine 3016 of ATM and initiating a phosphorylation cascade and DSB repair. This cascade activates the DNA repair pathway by phosphorylating H2AX (γH2AX), facilitating the recruitment of additional repair machinery. Tip60’s involvement extends to regulating cell-cycle arrest triggered by DNA damage, controlling the cell cycle through p53, and ensuring chromosomal stability during mitosis [34]. Tip60 also has an effect on the loosening of nucleosomes through interaction with the NuA4 complex members at DSB, resulting in an increase in DNA accessibility [14]. Many key proteins in the DDR, cell cycle, or chromatin remodeling pathways are regulated/acetylated by Tip60 (including ATM, H2AX, p53, Histones H4, and H2, Aurora B1, MRN, NuA4) [14,54,56,57,58,59,60,61,62,63]. Mutations that compromise cellular DDR pathways (including defects in the Tip60–ATM pathway) increase genomic instability and allow abnormal cell proliferation and tumor progression, ultimately significantly reducing patient survival [64,65]. In addition, Tip60-mediated genome instability is a feature of multiple diseases, including carcinogenesis, neurodegenerative diseases, aging, and immunodeficiency [66,67].

**Figure 3:**
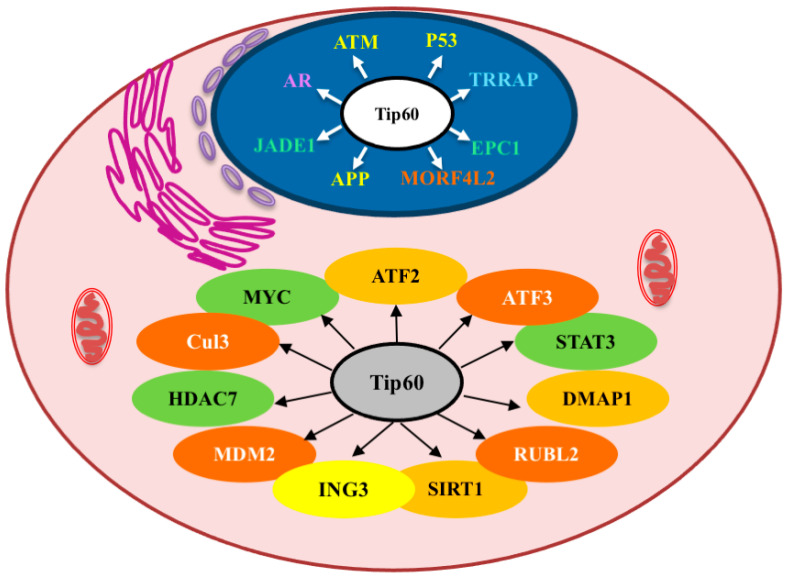
Selected Tip60-interacting proteins and their cellular localizations. Pink: cytoplasm; Blue: nuclear. The colors of the selected proteins shown relate to their processes, as indicated in Figure 1 (orange/dark orange: chromatin organization; green: transcription; yellow: metabolic stress response; purple: hormone response).

Interestingly, under “normal” non-tumorigenic conditions Tip60 is mainly found in the nucleus; however, Tip60 has been found to be strongly mislocalized to the cytoplasm in several cancers (Table 4) [68,69,70]. The effects of the mislocalization of Tip60 to the cytoplasm, and the consequences of its activity in cellular signaling while there, are poorly understood (Figure 3) but may underpin the novel pro-tumorigenic effects of Tip60 described (including simply the reduction of nuclear Tip60 levels, which inhibits its anti-tumorigenic activity).

Recently, it has been discovered that some KATs have additional catalytic activities, including Tip60 and p300, which display lysine isobutyrylation (Kibu) activity [128,129]. The post-translational modification Kibu has been shown on histones, where it regulates processes including metabolism (different metabolic pathways regulate the availability of acyl-CoAs required for different PTMs, such as Kibu) and transcription (through gene expression) [130,131]. As our understanding of the roles of Tip60 grows, due to improved understanding of its molecular functions, a clearer picture of its dysregulation and the consequences of this will be revealed.

## 7. Tip60 Regulation

Tip60 activity is regulated by multiple partners (Table 5) involving multiple mechanisms (including auto-acetylation, phosphorylation, SUMOylation), where these PTMs modulate the activity and role of Tip60 in processes like apoptosis induction. Tip60 auto-acetylation is a key regulatory mechanism regulating the DNA damage response, leading to ATM activation and the repair of double-strand breaks [132]. Additionally, it was shown that Tip60 is activated though phosphorylation by GSK3, which leads to p53-dependent apoptosis though the activation of p53 by Tip60 acetylation (of K120) [56,133]. Exploring the inhibitory mechanisms regulating Tip60 activity, it is known that the Abl kinase phosphorylates Tip60 (Y327), which indues association with FE65, inhibiting its HAT activity [134]. Furthermore, it has been shown that ATF2 (activating transcription factor-2) in conjunction with the Cul3 ubiquitin ligase, can regulate Tip60 activity (in DNA damage response signaling) by limiting the availability of Tip60, promoting its degradation [122]. To further highlight the complicated nature regulating Tip60 activity, in contrast to SIRT1-mediated deacetylation, HDAC3-mediated deacetylation extends Tip60’s half-life, mediating its availability and activity [109,135]. Interestingly, both HDAC3 and Tip60 can be localized in both the nucleus and cytoplasm, suggesting a potential stabilizing effect of HDAC3 on Tip60 [109]. Furthermore, it is likely that cell-type-specific regulation of Tip60 exists, further complicating our understanding of the effects of Tip60 regulation and the cellular effects on individual signaling pathways in each tissue.

## 8. Tip60 Tumor Profiling

Tip60 does not appear to act as a direct tumor suppressor or oncogene. Instead, it helps other proteins in these functions through its general acetyltransferase and transcriptional co-activator capabilities. This is demonstrated by the connection between Tip60 and p53 [56,133]. Interestingly, recent studies have shown a significant decrease in Tip60 expression in colon and lung carcinomas [33].

The involvement of Tip60 in cancer development is complex. As part of the multi-subunit NuA4 complex, Tip60 gets directed to target promoters by a variety of transcription factors. Operating within the NuA4 complex, Tip60 acetylates the nucleosomal histones H2A and H4, acting as a co-activator for the transcriptional factor. Furthermore Tip60 plays a key role in p53 activation, regulating apoptosis induction. Additionally, Tip60 is crucial for the expression of KAI1, a tumor suppressor in prostate cancer. Hence, the activity of Tip60 appears to rely on the specific context (cellular or molecular), and aberrations in lysine acetyltransferase activity can either promote or impede tumorigenesis in colon, breast, and prostate cancers [145]. Tip60 is downregulated in various cancers, such as colon, lung, breast, melanoma, prostate, gastric, lung, and pancreatic cancers. The hypothesis that eliminating the remaining Tip60 activity induces apoptosis has been confirmed, making Tip60 a promising candidate for targeted drug development as a lysine acetyltransferase inhibitor (KATi) [8,146]. A key feature of Tip60 is that its expression is essential for embryo viability [147,148], and it is vital for cell survival [149,150].

## 9. Tip60 Inhibitors

**Figure 4:**
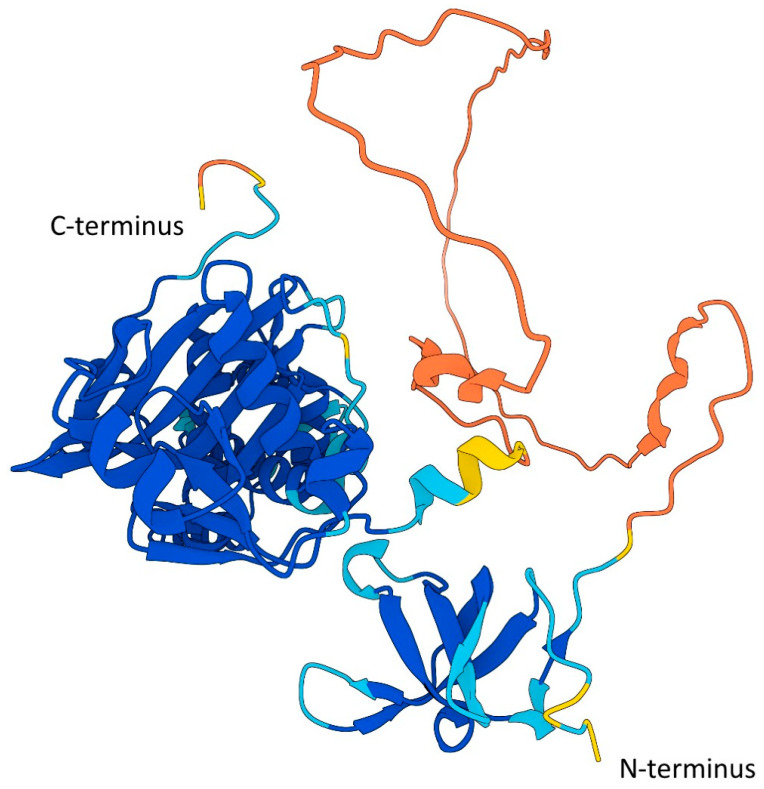
AlphaFold prediction of Tip60 structure. Based on Uniport accession A0A024R5E8 [155,156]. The region between 64aa and ~200aa has an expected position error of >30 Angstroms. Predicted template modeling (pTM) = 0.7, where a pTM score >0.5 indicates the overall prediction may be comparable to the true structure. Reside scoring—Blue: very high (pLDDT > 90); Aqua: high (90 > pLDDT > 70); Yellow: low (70 > pLDDT > 50); Orange: very low (pLDDT < 50).

The creation of Tip60-specific inhibitors (such as TH1834) has provided new tools for precise and tailored modulation of Tip60 activity, which are now used for thorough and specific molecular investigations of Tip60 activates to be explored [15,151,152,153]. The rationally designed method used to produce the Tip60 inhibitor TH1834 was facilitated by crystallization of the catalytic domain [8,101,150,154], and it is possible that future targeted inhibitors will make use of new advances in protein structure predictions by using the full protein (Figure 4) rather than just the crystalized catalytic acetyltransferase domain [155,156].

Recently, it has been clear that Tip60-targeting inhibitors show significant activities against cancer and other diseases [157] (Table 6). They are categorized into three groups: bisubstrate inhibitors, synthetic compounds, and natural compounds [7]. Among Tip60 inhibitors, some are well known (including Lys-CoA, anacardic acid, pentamidine, garcinol, and curcumin), but exhibit lower specificity affecting not only Tip60 but also pCAF and CBP/p300. However, several designed small molecules, including TH1834, NU9056, and MG 149, are selective for Tip60 [8,101,150,154].

## 10. Tip60 as a Biomarker

**Figure 5:**
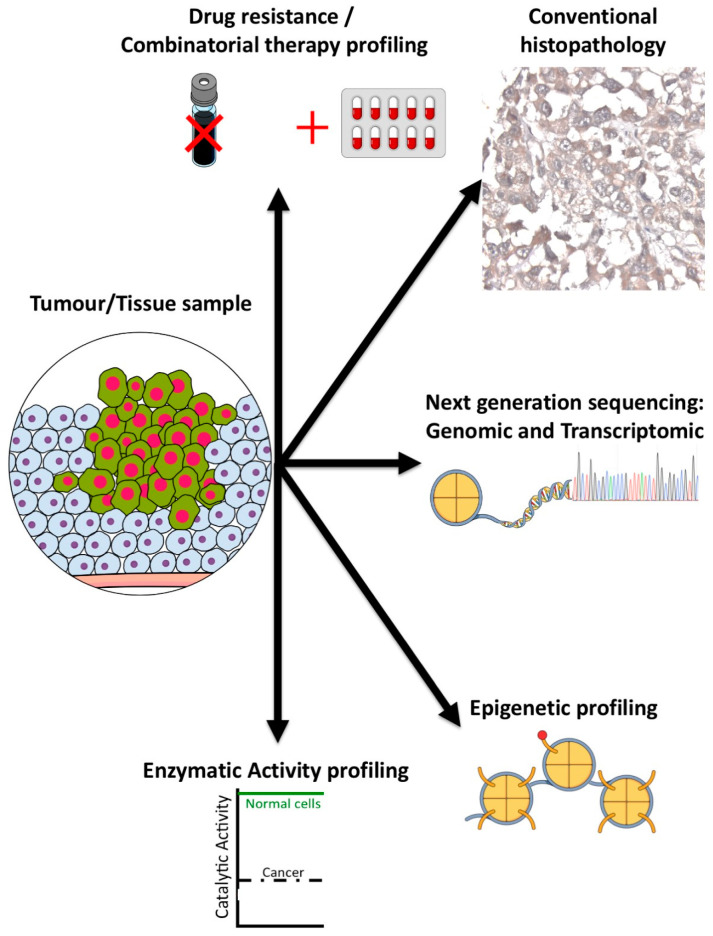
Tip60 biomarker profiling. Individual biomarker methods for evaluating Tip60 in tumors (methods as indicated).

To effectively utilize Tip60 inhibitors, specific paired biomarkers to identify cells with a sensitivity (or resistance) to these inhibitors are needed (Figure 5). As a key epigenetic and genome stability regulator, Tip60 (protein levels and/or activity) is itself a potential biomarker [68]. Levels of Tip60 have been investigated in several tumor types. In breast cancer, Tip60 transcript and protein levels have been found to be downregulated [68,149], while Tip60 was overexpressed in prostate cancer [101,168], and its activity upregulated in colon cancer [79] (Table 5). While mislocalization of Tip60, from the nucleus to the cytoplasm, has been observed in several cancer types (Table 7), the exact molecular consequences of this mislocalization remain to be fully elucidated.

As Tip60 has also been found to be dysregulated in other diseases, including neurodegenerative disorders, this raises the potential of using Tip60 as a biomarker in these conditions [174]. It has been found that in some neurodegenerative disorders, like Alzheimer’s disease (AD), histone acetylation by Tip60 in some loci is disrupted before amyloid-β accumulation. Detecting these spots could be an early biomarker for AD diagnosis and highlights the potential use of Tip60-targeting molecules as therapeutics in these diseases [174,175]. It has also been demonstrated that drugs inhibiting Tip60 activity may be useful agents for the treatment of ischemic heart disease [158].

## 11. Conclusions

Since Tip60 is an essential molecule with multiple cellular roles required for cell survival, more work is needed to better understand the individual (and linked) molecular roles it plays in normal cell types and tissues. The diverse molecular functions and roles of Tip60 make it a key new molecule for therapeutic targeting that has the potential to improve treatment in multiple diseases, ranging from cancer to neurological disorders. In addition, we need to improve our current understanding of the new (or misregulated) roles that mislocalized Tip60 plays in tumors or disease. Understanding Tip60 tissue-specific roles, and how tissue or disease-specific uses of Tip60 inhibitors vary is the key to facilitate the targeted use and clinical impact of Tip60 inhibitors in disease management.

## Figures and Tables

**Table 3:** Genes activated by Tip60.

Gene Name	Cell/Tumor Type Interaction Observed in	Reference
*TFF1*	Breast tumor	[36]
*Hoxa9*	Leukemia cell	[37]
*SRF*	Cardiac cells	[13]
*KAI1*	Prostate cancer cell	[38]
*GREB1*	Breast tumor	[36]
*Meis1*	Leukemia cell	[37]
*P21*	Osteosarcoma	[39]
*Fas*		[40]
*bax*		[40]
*Hdm2*		[40]
*POLQ*, *ASPM*, *EXO1*, *Gemin6*, *HESP1*, *KIF14*, *MTBP*, *PDSS1*, *TERF1*	Mammary tumors	[41]

**Table 4:** Tip60-interacting proteins and their subcellular localization.

Cancer Cells Lines/Model Used	Interacting Protein	Location	Reference
Hepatocellular carcinoma	TRRAP	n +	[26,71,72,73,74]
	P65		
	UHRF2		
	HDAC1		
	MYC		
	HDAC7	n/c ++	
	STAT3		
Colorectal cancer	TRRAP	n	[75,76,77,78,79,80]
	P400		
	RUVBL1		
	MRGBP		
	UHRF1		
	ATF3	n/c	
	TRCp120, BRD8		
Cervical cancer	TRRAP	n	[28,32,81,82,83,84,85,86,87,88,89,90,91,92,93,94,95,96,97]
	DNA-PK		
	P400		
	ANP32E		
	MRGBP		
	UHRF1		
	TRIM29		
	JAZF1		
	USP7		
	ING5		
	SIRT1		
	DNA-PKcs		
	ATM		
	P300		
	DMAP1		
	TRCp120		
	Nup62		
	SENP6/SUMO1	n/c	
	MDM2		
	IL-9 receptor		
	MYC		
Breast cancer	P53	n	[41,90,97,98,99,100]
	P400		
	RUVBL1		
	RUVBL2		
	Fe65	n/c	
	MORF4L1/MRG15		
	MYC		
Prostate cancer	P53	n	[101,102,103,104,105,106]
	TRIM68		
	MRGBP		
	AR		
	MORF4L1/MRG15		
	ING3	n/c	
	ATF2		
Lung cancer	UHRF1	n	[80,107,108,109,110,111,112,113]
	PXR		
	HDAC3	n/c	
	ATF3		
	RUVBL2		
	MDM2		
	Gas41/YEATS4		
	p14^ARF^		
Osteosarcoma/ chondrosarcoma	UHRF1	n	[32,80,92,107,111,114,115,116,117,118]
	MRGBP		
	RNF8		
	SOX9		
	ING5		
	SIRT1		
	DNA-PKcs		
	ATM		
	USP7	n/c	
	ATF3		
Blood cancer	MBTD1	n	[26,63,119,120,121]
	HDAC1		
	C/EBP α		
	STAT3	n/c	
	EPC2		
Melanoma	ING3	n/c	[105,122]
	ATF2		
Endometrial sarcoma	MEAF6	n	[123]
Renal cancer	JADE1	n	[124]
Neuroglioma cells	APP	n	[125]
Gastric cancer	KLF4	n	[126]
Esophageal squamous cell carcinoma	ATM	n	[127]
	FAM135B	n/c	

+ n: nucleus. ++ n/c: nucleus/cytoplasm.

**Table 5:** Tip60 regulation.

Tip60 (Protein) Regulatory Activity	Tip60 (Protein) Modification	Regulating Protein	Modified Tip60 Residues (aa)	Signaling Pathway
Activation	Phosphorylation	glycogen synthase kinase3 alpha/beta (GSK3A/GSK3B)	S86	Autophagy [136]
Activation	Phosphorylation	CDK1	S90	Receptor-mediated signaling processes/DDR [137]
Activation	Phosphorylation	p38 MAPK	T158	Oncogene-induced senescence [138]
Activation	Phosphorylation	DNA-PK	S199	DDR [139]
Suppression	Phosphorylation	Abl tyrosine kinase	Y327	DDR [134]
Suppression	Phosphorylation	PRKCE/PKCε	T298/S300	AR signaling [140]
Activation	Acetylation	Autoacetylation (Tip60)	K104	Apoptosis [132]
Activation	Acetylation	RUVBL1	-	Acetyltransferase activity of TIP60 [141]
Suppression	Acetylation, polyubiquitination	CBP/p300	K268	Apoptosis [142]
Activation	SUMOylation	UBE2I/Ubc9	K430, K451	DDR [95]
Activation	SUMOylation	PIAS4/PIASy	K430, K451	p53-induced autophagy [143]
Suppression	Negative effect on autoacetylation	SIRT1	K76, K80, K189, K327	DDR [144]
Suppression	Deacetylation	HDAC3	Unknown	Apoptosis [109]
Suppression	Downregulation of acetylation activity	RNF8	Unknown	DNA repair [115]
Suppression	Association	ATF2	aa 368–513	DDR [122]

**Table 6:** Specific Tip60 HAT inhibitors in pre-clinical studies.

Inhibitor Name	Effect	Disease	In Vitro/In Vivo	Reference
TH1834	Diminishing scarring, enhancing cardiomyocytes cell-cycle activating, reducing cardiomyocytes apoptosis, increasing cardiomyocytes density	Ischemic heart disease	In vivo (mice)	[158]
	Reducing tumor growth	Breast cancer	In vitro	[150]
			In vivo (mice)	[149]
	Altering expression of target genes related to cell proliferation and differentiation	Cataract	Ex vivo	[159]
	Suppressing tumor growth	Lung cancer	In vitro/ In vivo (mice)	[145]
	Increasing Foxp3 acetylation, enhancing Treg cell induction	Autoimmune disease, transplant	In vitro/ In vivo (mice)	[23]
NU9056	Inhibiting cellular proliferation, inducing apoptosis by activation of caspase 3 and 9, decreasing androgen receptor	Prostate cancer	In vitro	[101]
	Inhibiting the NLRP3 inflammasome, affecting gut microbiota	Cognitive impairment, emotional disorder	In vitro/ In vivo (mice)	[160]
	Inhibiting cell proliferation, inducing apoptosis, inhibiting the JAK2/STAT3 signaling pathway	Extra-nodal natural killer/T cell lymphoma	In vitro	[161]
	Shortening c-Myc half-life, downregulating miR-202 expression	Thyroid carcinoma	In vitro/ In vivo (mice)	[162]
	Reducing the ABCE1 protein acetylation, inhibiting the EMT, survival, migration, and invasion capacity of cancer cells	Esophageal cancer	In vitro	[163]
	Mediating the PI3K/AKT pathway	Allergic conjunctivitis	In vivo (mice)	[164]
	Inhibiting PfMYST, blocking *Plasmodium falciparum* growth and survival	Parasite *Plasmodium falciparum*	In vitro	[165]
	Decreasing the viability of KSHV-infected B lymphoma cells	KSHV-infected tumor	In vitro	[166]
	Increasing Foxp3 acetylation, enhancing Treg cell induction	Autoimmune disease, transplant	In vitro/ In vivo (mice)	[23]
MG 149	Inducing pro-inflammatory cytokines/chemokines, inhibiting cancer cells proliferation, inducing apoptosis in cancer cells	Malignant pleural mesothelioma	In vitro	[167]
	Decreasing the viability of KSHV-infected B lymphoma cells	KSHV-infected tumor	In vitro	[166]
	Increasing Foxp3 acetylation, enhancing Treg cell induction	Autoimmune disease, transplant	In vitro/ In vivo (mice)	[23]
	Inhibition of OXPHOS and mitochondrial biogenesis	Colon cancer	In vitro	[78]

**Table 7:** Tip60 or *Kat5* profiling in different tumor types.

Tip60 Dysregulation	Tumor	Experiment	Reference
Cytoplasmic mislocalization	Lung cancer	In vitro/in vivo	[145,169]
Downregulated Tip60, cytoplasmic mislocalization	Breast cancer	In vitro/in vivo	[68,149]
Downregulated Tip60 and *Kat5* transcript	Breast cancer	In vitro/in vivo	[41]
Overexpressed Tip60	Prostate cancer	In vitro/in vivo	[101,168]
Low KAT5 transcript expression	Prostate cancer	In vitro	[170]
Upregulation of Tip60 activity	Colon cancer	In vitro	[79]
Low KAT5 transcript expression	Melanoma	In vitro	[171]
Low KAT5 transcript expression	Cholangiocarcinoma	In vitro	[172]
Low KAT5 transcript expression	Gastric cancer	In vitro	[173]
